# Observations Suggested by Two Cases of Loss of Language

**Published:** 1846-11

**Authors:** Thomas Chambers

**Affiliations:** Late Physician to the Chelsea Dispensary


*Observations suggested by two Cases of Loss of Language.* By Thomas Chambers, M. D., late Physician to the Chelsea Dispensary.—Disease affects the intellectual powers in two ways, either by adding perverted function, or subtracting healthy function. On reviewing the classes of cases which come under these two categories, it will be observed that the causes of perverted action are usually to be sought in a part distant from the great centre of the mind's operations, while diminished or abolished action rightly leads us to suspect material change in that part. The unfounded fancies of the hypochondriac, and the excited imagination of the hysteric, refer us, by their names, under the ribs, or down in the pelvis, for information on their causes; in delirium traumaticum

> "the *pure* brain
> Does, by the idle comments that it makes,
> Foretell the ending of mortality."

In these cases from a distant cause, perverted function is added to the "pure" brain. On the other hand, when function is subtracted, we may usually conjecture some defect in the nervous centre; the deficiency of reasoning in the idiot is usually joined with malformation of the brain so marked as to affect the external appearance; "they have," as Fuller says, "heads either so small that there is not room for much wit, or so large that there is not wit enough for so much room." Now the manner in which another intellectual power, the memory, is affected, does, I think, sometimes serve as a means of diagnosis in certain obscure cases of cerebral disturbance which, from other symptoms, we do not know whether to ascribe to the slow supervention of delirium tremens or to chronic disease of the brain. The causes and cure of the former disorder are certainly not local. Now, under its influence the memory conveys impressions, but conveys them falsely; a man, for example, will remember that some well known crime has been committed, but remembers falsely that he himself was the perpetrator. Where, however, there is actual loss of memory, oblivion on some point, it may give a strong presumption that the mischief in the brain is more than functional. It did so in the following case, which, after this preface, I will transcribe.

Christian B., a German bootmaker, during the winter of 1842, used frequently to bring his sick child to me, and I often saw it at home, so that I had an opportunity of knowing his habits of mind and body. He was sober and intelligent, but a bad English scholar. In February, being disappointed in his hopes of success in trade, he became morose and dejected. At the beginning of March his wife came to me, stating that her husband had lost the use of his native language, but, what much surprised her, was still able to express himself in the little of ours that he knew. I did not take as a proof of this merely his not understanding my bad German, but could credit the report of his wife, who spoke it perfectly. This symptom was intermittent, as occasionally he seemed as capable of conversation, and as rational as usual. He complained, by signs, of pain in the head, and confusion of thought. The bowels were costive, the pulse rapid but soft, and the tongue creamy. There was a feverish heat of skin, but it was at all times moist, and occasionally bathed in copious perspiration. A rapid and trembling action of the hands in taking hold of anything, and a peculiar precipitate manner of putting out the tongue, bore a striking resemblance to what we see in delirium tremens. Though very morose and snappish in general, he was occasionally, without cause, joyous and easy, and always seemed glad to see me. There was no intolerance of light, and he was rather pleased with looking out of window. He was cupped, purged, and took small doses of mercury, and had cold lotions to the shorn scalp, with some slight benefit. The resemblance of the symptoms to delirium tremens I thought justified an attempt to remove the sleeplessness by morphia, but it did not appear beneficial. His wife, being in constant alarm for her children, had him removed to a lunatic asylum, where at first he seemed better, but, after six days, he suddenly became worse, and died so rapidly (apparently from asthenia,) that they had not time to inform his friends of his dangerous state before his death. The body was brought home, and, assisted by Mr. P. Hewett. I examined the head on the 23d of March. The subarachnoid cellular tissue of the vertex contained some clear fluid and the veins were somewhat congested. The substance of the brain was firm and healthy. The lateral ventricles contained no more fluid than is natural, but their membrane was slightly opaque. The arachnoid of the fourth ventricle had lost its glossy surface, and appeared as if sprinkled with fine grains of transparent sand : that also which covers the medulla oblongata was perhaps rather opaque. There appeared enough to justify us in attributing the man's death to inflammation of the arachnoid, and not to delirium tremens—an opinion which, before death, it would have been difficult to absolutely pronounce.

The peculiar symptom which caused me to relate this case, namely, the forgetfulness of his native tongue, whilst a strange one was retained, has at first a mysterious air. I think, however, we may in a great measure explain it by supposing that the more complex ideas, to express which he was accustomed to use the language he understood earliest and best, were lost; while the simpler and sensualler ones, which he could put into English, were retained. It was perhaps a partial lesion of memory, rather than a lesion of a peculiar class of ideas.

Many instances, however, are on record of such lesions of peculiar classes of ideas, while the rest of the intellectual faculties remain perfect; and, from the striking nature of these cases, they excite so much attention as to make them seem more common than they really are. The following was a remarkable one in point, where, from other symptoms, there was no doubt that there was material change in the brain itself, though the confirmation by actual inspection was wanting.

Harriet C., æt. 12, had typhus fever in December, 1845 ; she had much delirium and low symptoms, but, as is usual with children, soon got about again, and was able to return to school. However, after a few days' attendance, she was one evening, on returning thence, taken with a fit, of an undecided epileptic character, had rigors, and was again delirious. The delirium was monotonous, and remarkable for her constant repetition of the word "sinner" with every variety of intonation. Wine and bark were, as during her former attack, resorted to, but symptoms of slight effusion in the brain caused its suspension. She recovered after a few weeks so as to be up and dressed, but with the loss of power to pronounce any word except the one she had so often repeated during her fever. This she made serve to express all her ideas; for denial she shook her head and said "sinner;" assent was expressed by the same word, and bread and butter was called "sin-un-sinner." She perfectly understood all that was said to her, and appeared capable of reading her usual lessons. Blisters were applied behind her ears, and small doses of mercury administered, and at the same time her mother and family were instructed to teach her as they would an infant to talk. I also took opportunities of showing her, by exaggerated motions of my mouth and throat, the way of forming the letters, in the manner in which the born deaf and dumb are instructed, and found her intelligent and ready. She soon acquired the word "yes," and other elementary expressions, and by the end of the spring was able, as her mother told me, "to talk like an old woman." Symptoms of consumption had, however, appeared, and she died this last summer under the care of another medical man, whose kind efforts to obtain a post-mortem examination for me were unavailing.

The instances usually cited of the loss of memory on special subjects are where it has been the consequence of blows or some such external injury to the head; still both it and general loss of memory will occasionally follow typhus fever. In some epidemics it has done so with such marked frequency as to form one of the characteristics of the prevailing disorder. This was the case in the great plague of typhus which followed the famine at Athens in the Dorian war, as we learn from Thucydides (book ii. cap. 49,) where he tells us that of those who recovered, some entirely lost the recollection of their former associates, and some even the idea of their own personal identity. The symptom is an unfavorable one at all times, as showing that material mischief is done to the brain, but it is much less unfavorable when a consequence of fever, than where, as in the case of the German bootmaker lately cited, it commences the illness. The history I have related is sufficient to show it to be curable.—*London Medical Gazette.*

